# Observed Changes in Risk during Naturopathic Treatment of Hypertension

**DOI:** 10.1093/ecam/nep219

**Published:** 2011-03-13

**Authors:** Ryan Bradley, Eva Kozura, Jennifer Kaltunas, Erica B. Oberg, Jeffery Probstfield, Annette L. Fitzpatrick

**Affiliations:** ^1^Bastyr University, Kenmore, WA 98028, USA; ^2^University of Washington, Institute for Translation Health Sciences, Seattle, USA; ^3^University of Washington, Department of Epidemiology, Seattle, WA 98195, USA; ^4^University of Washington, Department of Cardiology, Seattle, WA 98195, USA

## Abstract

Few outcome assessments are published from complementary and alternative medicine (CAM) practices. We aimed to describe patient and practice characteristics of ND care for hypertension (HTN), quantify changes in blood pressure (BP), and evaluate the proportion achieving control of HTN during care. A retrospective, observational study of ND practice in HTN was performed in an outpatient clinic in WA State. Eighty-five charts were abstracted for the final analysis. At initiation of care, the mean patient age was 61 years, with 51% having stage 2 HTN, despite common use of anti-hypertensive medications (47%). Patients with both stage 1 and stage 2 HTN appeared to improve during care, with stage 2 patients achieving mean reductions of −26 mmHg (*P* < .0001) and −11 mmHg (*P* < .0001) in systolic BP (SBP) and diastolic BP (DBP), respectively. The proportion of patients achieving control (<140/90 mmHg) in both SBP and DBP was increased significantly from 14 to 44% (*P* < .033), although the statistical significance was not maintained upon correction for multiple comparisons. BP appears to improve during ND care for HTN, in a high-risk population. Randomized trials are warranted.

## 1. Introduction

Hypertension (HTN) is an important, poorly controlled risk factor for developing cardiovascular disease (CVD) in the USA, including myocardial infarction and stroke [1]. Data from the Third National Health and Nutrition Examination Survey (NHANES) suggest that patients with uncontrolled HTN have nearly a 2-fold risk of cardiovascular mortality compared with normotensive adults [2]. The Seventh Report of the National Committee on Prevention, Detection, Evaluation and Treatment of High Blood Pressure (JNC-7) suggests that risk for CVD event doubles with each 20 mmHg increment of systolic blood pressure (SBP), and each 10 mmHg increment of diastolic blood pressure (DBP), over 115/75 mmHg [3]. Despite evidence of increased utilization of medical care for HTN, large longitudinal cohorts suggest that up to 50% of all US patients with HTN are not at their optimal treatment target [4].

Intensive lifestyle counseling recommendations are included in most major guidelines for HTN management, including JNC-7; however, the available evidence suggests these recommendations are infrequently given to patients [3, 5]. According to the National Ambulatory Medical Care Survey, fewer than 50% of patients with HTN received lifestyle counseling [6]. In contrast, descriptions of naturopathic (ND) practice suggest clinical recommendations by ND physicians include diet counseling, exercise prescription and stress management advice for 69–100% of diabetes patients [7–9].

In Washington State, ND physicians are primary care providers with a scope of practice that includes nutritional supplementation, herbal medicine, nutrition, exercise, physical medicine modalities, minor surgery and most prescription drugs, including all classes of anti-hypertensive medications. Frequently classified as “complementary and alternative medicine (CAM)”, ND medicine is a variant of healthcare delivery that provides an interesting model to study the effectiveness of cardiovascular risk factor reduction because it includes health promotion counseling, nutritional supplementation *and* pharmacologic treatment options. ND care creates a laboratory for evaluations of both the effectiveness of health promotion in practice and of an “integrative” practice model, including CAM plus select prescription therapy.

Few data are available regarding the effects of CAM practice in the treatment of HTN; however, national surveys suggest CAM use in patients with CVD is common. Specifically, data from NHANES 2002 suggests that nutritional supplement use amongst patients with HTN is commonplace; 62% of respondents used some form of nutritional supplementation, with multivitamins (40%), antioxidants (26%) and calcium (27%) being the most common [10]. Practice-based evaluations noted that 58% of patients using nutritional supplements were simultaneously taking medications with narrow therapeutic windows including digoxin and warfarin, reinforcing the need for practice evaluations of outcomes [11]. ND practice evaluations in type 2 diabetes have reported mean BP reductions, and recommendations for nutritional supplementation in 100% of patients [8]. Unfortunately, “whole systems” evaluations of HTN treatment from unique CAM disciplines have not been reported in the medical literature, leading to the discouragement of CAM use for HTN by some authors [12]. Here, for the first time, we provide a quantitative evaluation of “whole practice” ND clinical care for the treatment of HTN, including a description of nutritional supplementation use in practice and estimates of change in clinical risk.

## 2. Methods

In order to describe ND practice for the treatment of HTN, and to estimate possible changes in cardiovascular risk, we performed a retrospective, observational study of HTN care in an outpatient ND clinic, the Bastyr Center for Natural Health, in Seattle, WA. We aimed to describe the patient population pursuing ND care for HTN, describe practice characteristics including the delivery of health promotion counseling and nutritional supplementation, and estimate the changes in SBP and diastolic DBP including the proportion of patients who achieve controlled BP during care. The study was reviewed and approved by institutional review boards at both Bastyr University and the University of Washington.

### 2.1. Case Inclusion and Data Collection

Medical charts were identified through clinic scheduling software, searchable by ICD-9 code, and data were abstracted between December 2006 and June 2007. The analyses included patients meeting three *a priori*-specified inclusion criteria: (i) an ICD-9 assessment of HTN was made by the ND (i.e., 401.XX–405.XX), (ii) evidence of at least 6 months of ND care occurred between 2001 and 2006 and (iii) ND care provided specifically for HTN (versus accompanying symptoms or associated conditions). A 6-month duration of care was specified in an attempt to balance bias between using either too short or too long of an observation period. Specifying too long of a care period may oversample the uniquely motivated, whereas too short of a care period may underestimate effects of a re-iterative care process.

The following data were collected: patient characteristics including race, gender, age and current anti-HTN medications; care characteristics including primary versus adjunctive care, duration of care, presence of “care gaps” (interval in appointment dates greater than 6 months apart), and number of visits during the care period; BP measurements over the duration of care; and characteristics of treatment recommendations, including lifestyle counseling (diet and exercise), nutritional supplementation and the initiation of new anti-hypertensive prescription medication. “Primary care” (PCP) status was defined as either the ND being designated on provider contact sheet as the PCP, or ND provision of annual preventive service visits. Care characteristics were calculated as the percentage of patients receiving various recommendations, including specific dietary recommendations, exercise prescription and nutritional supplementation, as well as the percentage of visits during which these recommendations were given.

### 2.2. BP Measurement and Definition of Control

BP data from the patient's first visit was compared with the most recently available measurement at the time of chart abstraction in analyses. The statistical significance of mean BP changes was determined using two-tailed, paired *t*-tests of homogeneity applied to the difference between the most recently available reading, and the baseline reading, with our null hypothesis being the change in BP was zero between these time points. These tests were applied to the BP data independent of stage, and then repeated after stratification by stage. Patients were assigned to stages based on JNC-7 criteria (stage 1 = SBP 140–159 mmHg or DBP 90–99 mmHg; stage 2 = SBP ≥ 160 mmHg or DBP ≥ 100 mmHg) [3]. In addition, the proportion of patients in “good BP control” at their first visit was calculated and compared with the proportion in control at their most recent measurement, using Fisher's exact test. “Control” was defined as <140/90 mmHg at either point (baseline versus last observed). In addition, the proportions of patients in systolic *or* diastolic control, and the proportion in systolic *and* diastolic control, were calculated and compared. Following data abstraction and coding, analyses were performed on Statacorp STATA for Mac v. 10.0.

## 3. Results

### 3.1. Final Study Sample

After applying inclusion criteria to all records for patients receiving care for HTN between 2001 and 2006 (*n* = 249), a total of 85 charts were included for detailed data abstraction. The most common reason for exclusion was a lack of a 6-month duration of care (excluded *n* = 121 or 49% of total); this finding is consistent with the high degree of adjunctive ND care use. Baseline characteristics of this sample population are reported in Table 1. As expected, baseline SBP was crudely associated with continuous age ( *P*_crude._ = 0.44; 95% CI: 0.17, 0.71, *P* = .002), male gender (*β*_crude_ = 8.66 (95% CI: 0.08, 17.2) for male versus female, *P* = .05) and continuous baseline diastolic BP (*β*_crude_ = 0.61; 95% CI: 0.27, 0.96, *P* = .001), but not race (*β*_crude._ = 0.35; 95% CI: − 4.4, 5.1 for Caucasian versus non-Caucasian, *P* = .88).

### 3.2. Characteristics of Care Delivery and Treatment Recommendations

Table 2 summarizes select characteristics of ND care for HTN. On average, patients attended 8.7 ND visits over a 13.8 (±8.7 months) care period. Thirty-two patients (37.6%) had evidence of at least one 6-month gap in their ND care. ND care remained mostly adjunctive (76.5%); however, 23.5% of patients appeared to utilize ND care as primary care. The provision of diet, exercise and preventive counseling was common during ND care for HTN. Eighty-three patients (97.6%) received dietary counseling, reiterated over 54.8% of all visits. Fifty-eight patients (68.2%) received counseling to increase physical activity, reiterated over 33% of all visits. Specific dietary recommendations are also summarized in Table 2. In nearly all cases, patient educational handouts were evident in the medical record.

**Figure 1:**
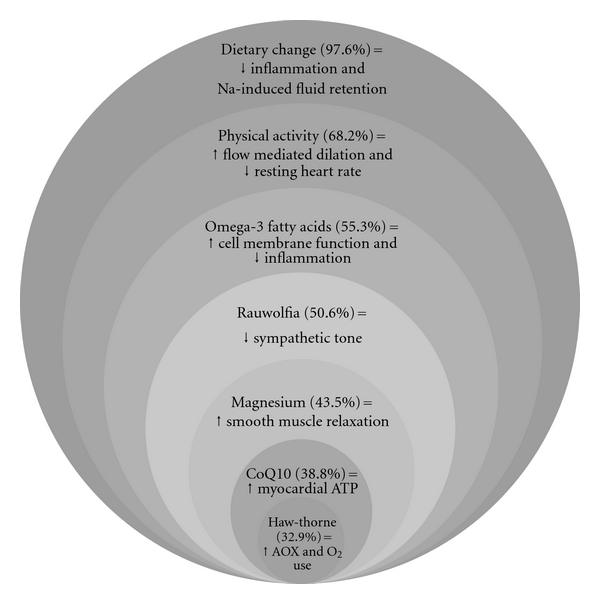
Hypothetical model for select elements of naturopathic treatment of hypertension. Values in parentheses indicate the percentage of patients receiving this recommendation. Citations for the hypothetical actions are included in the main text. AOX, antioxidant action; ATP, adenosine triphosphate; Na, sodium; O_2_, oxygen.

As shown in Table 2, the prescribing of nutritional supplementation was also typical in ND treatment of HTN; all patients were recommended to take nutritional supplements. Omega-3 oil from fish was the most commonly recommended supplement (55.3%), followed by “Combo 1”, a commercial botanical/mineral combination containing *Rauwolfia, Arjuna, Convolvulus*, *Tribulus* and magnesium aspartate (50.6%); magnesium (43.5%); CoQ10 (38.8%); *Crataegus* (hawthorne) (32.9%); “Combo 2” containing vitamin B6, magnesium citrate-malate, *Allium sativa*, *Taraxacum*, *Rauwolfia*, *Crataegus*, *Leonurus*, *Passiflora*, Resveratrol and CoQ10 (12.9%); and potassium (8.2%). Concurrent prescribing of these supplements was common (42%). Many other nutritional and botanical supplements were recommended; however, these products were recommended to <5% of patients.  Figure 1 presents the hypothetical rationale for elements of health promotion counseling, as well as for select nutritional and herbal medicines [13–19].

### 3.3. Observed Changes in BP

**Figure 2:**
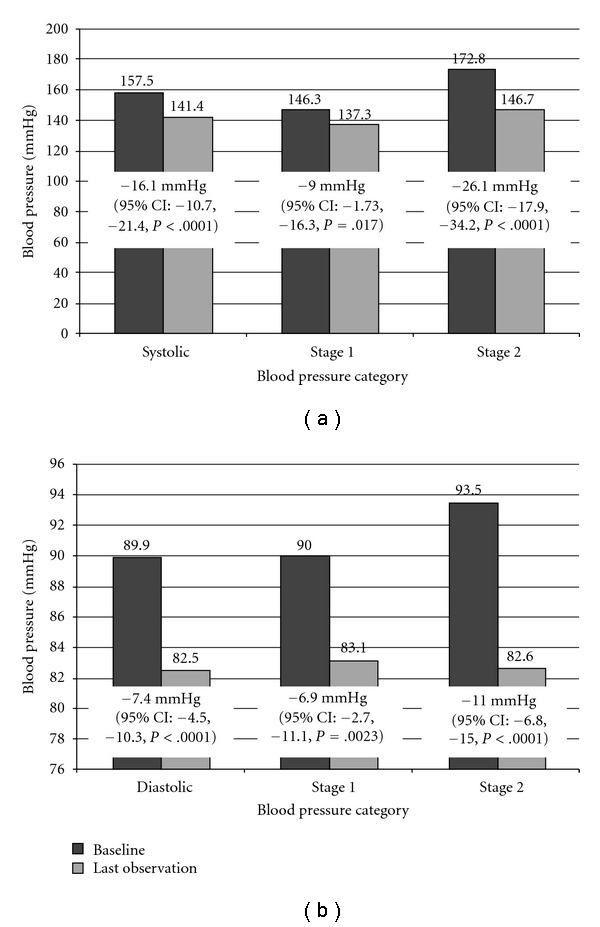
Mean changes in (a) systolic blood pressure and (b) diastolic blood pressure observed during ND care. Last observed BP—baseline BP, that is, negative values are reductions. Paired two-sided *t*-tests for homogeneity, H_0_ = change = 0. Bold indicates statistical significance after correction for multiple comparisons.

Figures 2(a) and 2(b) detail the average change in SBP and DBP during the course of ND care, combined and by baseline stage. As the figures demonstrate, BP reductions were sizable, statistically significant based on *t*-tests for homogeneity and positively correlated to stage at baseline. Patients with both stage 1 and stage 2 HTN appeared to improve during care, with stage 2 patients achieving mean reductions of −26 mmHg (*P* < .0001) and −11 mmHg (*P* < .0001) in SBP and DBP, respectively.

Table 3 compares the proportion of patients in good BP “control” at their first visit, compared with control at their most recent measurement during ND care. We found a strong trend for both an increased proportion of patients in *either* systolic *or* diastolic control, and an increased proportion in *both* diastolic *and* systolic control. Notably, only 14% were in SBP and DBP control at baseline, versus 44% at the last available measurement. These findings, in combination with sizable average SBP and DBP reductions in both stages, suggest that clinically meaningful reductions are achieved during ND care.

Given the marginal statistical significance of several of the results, the statistical significance would not hold up to a Bonferroni-corrected significance threshold (*n* = 9; *P*_corr._ < .0056); however, mean reductions for all categories, except stage 1 SBP, would hold up this corrected significance threshold.

## 4. Discussion

The results of this study suggest that BP is significantly reduced during ND care and the reductions appear clinically meaningful (−16.1 mmHg mean reduction in SBP, *P* < .0001 and −7.4 mmHg mean reduction in DBP, *P* < .0001). Both severe (stage 2) and moderate (stage 1) HTN appear to improve. These findings compare to recent meta-analyses of typical reductions in BP from pharmaceutical anti-HTN medications in which mean reductions in SBP of −15 mmHg are seen, on average, per class of medications [20]. Forty-four percent of patients achieved new BP control during ND care. Notably, the proportion of patients in *both* systolic *and* diastolic control to <140/90 *tripled* from 14.2 to 43.5%, or an increase of 29.3%. This finding compares to meta-analysis of 63 trials of primary care HTN quality improvement in which the increase in the proportions of patients reaching SBP and DBP targets were 16.2% (interquartile range 10.3–32.2) and 6.0% (interquartile range 1.5–17.5), respectively [21]. Despite not holding up to a corrected significance threshold, the trend is strong and the observed point estimates are clinically meaningful.

In addition, our study suggests that the dietary advice given by ND physicians is evidence based. Recommendations to increase dietary fish (20%), or take fish oil (55.3%), are supported by the American Heart Association; however, literature suggests that this recommendation is infrequently given in standard primary care (17%) [22, 23]. Additional recommendations include sodium reduction and increasing whole grains, legumes, fish, fruit and vegetable consumption. When adopted, the dietary recommendations have the potential to reduce patient risk for multiple chronic diseases, not just HTN [24].

Supporting the notion that ND care is a laboratory for studies of the delivery of health promotion counseling in clinical practice, we observed recommendations for dietary change and increased physical activity were commonplace in ND care for HTN (Table 2) and may be responsible for some, or all, of the observed changes. The observed reductions are comparable to the contribution of lifestyle modification on BP reductions reported in JNC-7 guidelines [3]. Estimates for the delivery of health promotion counseling to at risk patients (68.2–97.6%) exceed estimates from routine allopathic care and are consistent with American Heart Association and the US Preventive Services Task Force (USPSTF) guidelines [5, 6, 25].

This study has several limitations. While the observed changes are concurrent with ND care, causation cannot be determined from observational data. This study lacks a natural history control group; however, advancing age and higher baseline BP were both predictors of *not* reaching BP control in the Framingham cohort, suggesting elevated BP typically does not self-resolve in a population of advancing age [26]. Our study may not have accounted for all healthcare services utilized; it remains unknown whether specific ND treatments, including supplements, contributed to the BP reductions or if other recommendations, including medication adherence reminders or the care of other providers, resulted in these changes. In the 29% of patients receiving advice to take new anti-HTN medications, subsequent multivariate logistic regression analyses showed no association with either baseline or new anti-HTN medications and odds of control (data not reported here). Also the population under study was self-selecting ND care, with a minimum of 6 months of care, and therefore these patients are likely exceptionally motivated for health improvement. Although multiple testing increased the probability of a type 1 error, we have discussed the results in relation to corrected significance thresholds. Finally, despite its importance in predicting risk for CVD and CVD-related mortality, BP remains a surrogate outcome measure, and therefore the impact of the observed reductions on clinical cardiovascular events or other health outcomes cannot be determined [1, 27].

The results of our study, although preliminary, have important clinical implications. Specifically, risk reduction from HTN appears to occur during ND care. The observed *tripling* of the proportion of patients in HTN control to <140/90 mmHg is both encouraging and provocative especially given the advanced age and significant proportion with stage 2 HTN. It has been reported that patients who use CAM trend toward lower adherence to medications and therefore our study offers some reassurances that NDs appear to engage on HTN clinically, offer evidence-based health promotion counseling and prescribe anti-HTN medications as needed to reduce risk [28]. The observed greater reductions in those with stage 2 HTN at the beginning of care, compared with those with stage 1, is also reassuring and suggest ND treatment is either effective at various risk stages and/or that treatment is tailored depending on risk at presentation. Nutritional supplementation was used routinely in patients with HTN, which despite hypothetical effects (see Figure 1) have limited data supporting clinical use. Exceptions include omega-3 fatty acids from fish (for which two meta-analyses suggest benefit in HTN) and CoQ10 (which has been recognized as having “sound” evidence) [29–34].

Future studies of the impact of ND and other CAM services on HTN and composite cardiovascular risk should include prospective, observational evaluations of multiple ND practices, allowing for multivariate analyses in larger sample sizes to reduce the risk of type 2 error; these evaluations could be best accomplished with practice-based research networks initiated through academic and large group ND practices. Given the costs and unknown medication interactions of supplementation for HTN, prospective monitoring and factorial clinical trials on the use of supplement combinations should also be pursued.

## 5. Conclusion

Reductions in BP were observed during care by ND physicians; however, the care characteristics responsible for the changes could not be determined from this analysis. ND practice appears to have several positive qualities from a public health perspective, including clinical engagement on CVD risk factor reduction, a high prevalence of guideline recommended lifestyle counseling and the use of evidence-based dietary recommendations.

## Funding

This research was supported by the National Center for Research Resources (NCRR) (1KL2RR025015-01), a component of the National Institutes of Health (NIH) and the NIH Roadmap for Clinical Research.

## Figures and Tables

**Table 1:** Characteristics of sample population at initial ND visit for HTN.

Population characteristic (*n* = 85)	Mean (SD) or *n* (%)
Age (years)	60.6 (14.9)
Sex	
Male	35 (41%)
Female	50 (59%)
Ethnicity	
White	45 (53%)
Non-White	12 (14%)
Unknown	28 (33%)
BP (mmHg)	
SBP	157.5 (20)
DBP	89.9 (11.8)
BP stages	
Stage 1	30 (35%)
Stage 2	43 (51%)
Controlled (<140/90) on anti-HTN medications	12 (14%)
Use of anti-HTN medications at first visit	40 (47%)
ND prescribed new anti-HTN medication added during ND care period	14 (16%)
Total new anti-HTN medication added during ND period	25 (29%)

**Table 2:** Care characteristics of ND care for HTN.

Care characteristic	Mean (mode, median and IQR) or *n* (%)
Number of visits	8.7 (4,7,4)
Duration of care (months)	13.8 (7,12,8)
Presence of care “Gap”	32 (37.6%)
Primary care	20 (23.5%)
Category of health promotion advice	*n* (%) receiving advice
Diet	83 (97.6%)
Exercise	58 (68.2%)
Alcohol	48 (56.5%)
Tobacco	40 (47.1%)
Specific dietary advice	*n* (%) receiving advice
Increase fruit and vegetable intake	71 (83.5%)
Increase legumes/beans/nuts/whole grains	43 (50.6%)
Reduce dietary sodium	36 (42.3%)
Increase dietary fiber	31 (36.5%)
Increase fish intake	17 (20%)
Adopt the “DASH” diet	16 (18.8%)
Adopt the “Mediterranean” diet	3 (3.5%)
Nutritional supplementation	*n* (%) receiving recommendation
Omega-3 Oils from Fish	47 (55.3%)
“Combo 1”	43 (50.6%)
Magnesium	37 (43.5%)
Coenzyme Q10	33 (38.8%)
*Crataegus oxycanthus* (Hawthorne)	28 (32.9%)
“Combo 2”	11 (12.9%)
Potassium	7 (8.2%)

IQR: Inter-quartile range.

**Table 3:** Patients with controlled BP during ND care compared with baseline.

“Control” definition	*n* (%) at baseline	*n* (%) at last observation	*P*-value^a^
SBP < 140 mmHg	14 (16.5%)	43 (50.6%)	.038
DBP < 90 mmHg	35 (41%)	61 (71.8%)	.026
Combined systolic and diastolic control			
Neither SBP *nor* DBP < 140/90 mmHg	48 (56.5%)	18 (21.2%)	.033
Either SBP *or* DBP < 140/90 mmHg	25 (29.4%)	30 (35.3%)	
Both SBP *and* DBP < 140/90 mmHg	12 (14.2%)	37 (43.5%)	

^a^Fisher's exact test.
